# The value of ^18^F-FDG PET/CT imaging in predicting the efficacy of PD-1/PD-L1 immune checkpoint inhibitors in advanced non-small cell lung cancer

**DOI:** 10.3389/fonc.2025.1651843

**Published:** 2025-12-01

**Authors:** Huiwen Luo, Linlin Li, Jian Wang, Xuefeng Hou, Xiaofeng Li, Wengui Xu, Wen Zhou, Dong Dai

**Affiliations:** 1 Health Management Center, Tianjin Medical University Cancer Institute and Hospital, National Clinical Research Center for Cancer, Tianjin, China; 2 Tianjin’s Clinical Research Center for Cancer, Tianjin, China; 3 Key Laboratory of Cancer Immunology and Biotherapy, Tianjin Medical University, Ministry of Education, Tianjin, China; 4 Department of Molecular Imaging and Nuclear Medicine, Tianjin Medical University Cancer Institute and Hospital, National Clinical Research Center for Cancer, Tianjin, China; 5 Department of Medical Imaging, Tianjin Children's Hospital (Tianjin University Children’s Hospital), Tianjin, China; 6 Tianjin Key Laboratory of Technologies Enabling Development of Clinical Therapeutics and Diagnostics, School of Pharmacy, Tianjin Medical University, Tianjin, China

**Keywords:** ^18^F-FDG, PET/CT, NSCLC, immune checkpoint inhibitor, prognosis

## Abstract

**Background:**

We aimed to investigate the relationship between metabolic parameters based on ^18^F-FDG PET/CT and the prognosis of programmed cell death protein 1 or its ligand (PD-1/PD-L1) Immune checkpoint inhibitors (ICI) with/without chemotherapy in advanced non-small cell carcinoma (NSCLC).

**Methods:**

In this single-center retrospective study, 115 patients with advanced NSCLC who underwent PD-1/PD-L1 ICI with/without chemotherapy with baseline ^18^F-FDG PET/CT between 2018 and 2023 were enrolled. Baseline clinical parameters, PD-L1 gene expression, and hematological parameters (including derived neutrophil-to-lymphocyte ratio (dNLR) and lactate dehydrogenase (LDH)) were collected. The optimal cutoff value of PET metabolic parameters was obtained using the receiver operating characteristic (ROC) curve. We used the Chi-square test, binary logistic regression, and cox-regressive analysis to analyze the relationship between the above parameters and clinical outcomes, including disease clinical benefit (DCB), progression-free survival (PFS), and overall survival (OS).

**Results:**

A total of 115 patients were enrolled in the group. After univariate and multivariate analyses, MTVwb [P = 0.002, HR 3.906 (3.644 - 9.279)] and treatment modality [P < 0.001, HR 8.11 (2.51 - 26.21)] were identified as independent prognostic factors for DCB. MTVwb [P = 0.012, HR 2.041 (1.173-3.552)] and treatment modality [P = 0.030, HR 2.045 (1.073 - 3.896)] are independent prognostic factors for PFS, while MTVwb [P =0.013, HR 4.173 (1.356-12.842)] is an independent influencing factor for OS. In the immunotherapy combination group, there was a significant correlation between PD-L1 expression and TLGwb (p*=*0.029).

**Conclusions:**

Among patients with advanced NSCLC who received PD-1/PD-L1 ICIs in combination or without chemotherapy, MTVwb was an independent influencing factor for DCB, PFS, and OS. The treatment modality is also an independent influencing factor for DCB and PFS. ^18^F-FDG PET/CT has a very promising application prospect in predicting the prognosis of patients treated with immune checkpoint inhibitors. At the same time, the level of sugar metabolism is positively correlated with PD-L1 expression.

## 1 Introduction

On February 2, 2024, the latest “Global Cancer Burden Growing and Mounting Need for Services” (1)released by the International Agency for Research on Cancer (IARC) of the World Health Organization showed that lung cancer is the most common type of cancer in the world, and ranks first in cancer “death list”. Non-Small Cell Lung Cancer (NSCLC) accounts for about 85% of all types of lung cancer (2) In recent years, immune checkpoint inhibitors (ICI), especially programmed death receptor 1 (PD-1) and its ligand 1 (PD-L1), have been widely used.PD-1/PD-L1 inhibitors have significantly improved the treatment of patients with advanced NSCLC (3). However, in view of the occurrence of pseudoprogression, hyperprogression, and withdrawal of immune-related adverse reactions in clinical practice, as well as the heterogeneity of response to immunotherapy among different NSCLC patients, we should choose an effective indicator to predict the survival prognosis of NSCLC patients before treatment. Given the occurrence of phenomena such as pseudo-progression, super-progression, and discontinuation of immunotherapy due to immune dysfunction in clinical practice, as well as the heterogeneity of responses to immunotherapy among different NSCLC patients. Selecting an effective indicator for predicting the survival prognosis of NSCLC patients before treatment is of vital importance for screening out the sensitive NSCLC patients for immunotherapy and improving the overall treatment efficacy. This is because it can help to identify patients who are more likely to respond to immunotherapy and thus enhance the effectiveness of the treatment (4).Furthermore, even among those patients who initially responded to ICIs, eventually, they will develop resistance to ICIs. This may require an effective combination with other treatments to effectively remodel the immune microenvironment into a mode more suitable for immunotherapy (5). A large number of studies have confirmed that immunotherapy combined with chemotherapy can play a synergistic effect, which can benefit patients more than chemotherapy alone (6, 7).

PD-L1, measured using immunohistochemistry, is the only approved biomarker used to select patients who can benefit from immunotherapy (8). However, a large number of studies have found Tumor Mutational Burden (TMB) (9, 10), derived neutrophil to lymphocyte ratio (dNLR) (4), serum tumor markers (such as encompass carcinoembryonic antigen (CEA), neuron-specific enolase (NSE), cytokeratin fragment 21-1 (Cyfra21-1) and carbohydrate antigen (CA19-9) (11)have predictive significance. Among them, dNLR includes monocytes and other granulocyte subsets, which can capture systemic inflammatory responses and changes in peripheral blood white blood cells. But these parameters provide limited prognostic information at the individual level. Therefore, effective biomarkers for predicting the efficacy of ICIs are still being explored. ^18^F-FDG PET/CT imaging, as an important clinical imaging examination method, has been widely applied in clinical practice for cancer patients. It plays a significant role in the clinical diagnosis, staging, treatment evaluation, and prognosis of NSCLC patients. At the same time, relevant studies have also confirmed that ^18^F-FDG PET/CT metabolic parameters can reflect the level of tumor glucose metabolism and the state of the tumor immune microenvironment to a certain extent (12).

This study will focus on discussing the application value of PET metabolic parameters and the aforementioned related hematological indicators in the prognosis of survival of advanced NSCLC patients who received PD-1/PD-L1 ICIs in combination or without chemotherapy. It aims to seek an effective PET/CT imaging indicator for NSCLC patients to screen out the sensitive population before treatment.

## 2 Materials and methods

This research plan has been reviewed and approved by the Institutional Review Board (IRB) of Tianjin Medical University Cancer Hospital (Approval Number: [bc20240732]). Given that this study is a retrospective analysis, all data have been de-identified and the identities of individual patients cannot be identified. Moreover, the research risk is no greater than the minimal risk. The committee has approved the exemption from obtaining informed consent from the patients.

### 2.1 Patients selection

A total of 115 patients with advanced NSCLC (stage III-IV) in our center were enrolled in this retrospective study. These patients started receiving PD-1/PD-L1 ICIs with/without chemotherapy between January 2018 and June 2023, and baseline ^18^F-FDG PET/CT images and blood sample data were collected. Inclusion criteria were as follows: 1. PET/CT was performed before immunotherapy alone or chemotherapy combined with immunotherapy;2.NSCLC was diagnosed by fine needle aspiration or postoperative pathology (The clinical stage was III or IV); 3. Complete and traceable medical records (to include the clinical data of enrolled patients, including age, gender, smoking status, pathological type, previous treatment history, etc.); 4.^18^F-FDG PET/CT was performed <60 days before starting immunotherapy, and a time span of up to 3 months was allowed if no tumor-specific therapy was applied during this period;5. The inclusion criteria for blood sample evaluation before ICIs treatment was 28 days; Exclusion criteria: 1. Previous history of other tumors; 2. Patients with brain metastases (The delineation of target volume has a large influence factor). Patient demographic data, disease characteristics, hematologic parameters, treatment, and clinical outcomes were reviewed.

### 2.2 Therapy methods

In this study, patients were treated with PD-1/PD-L1 immune checkpoint inhibitors in combination with or without chemotherapy. Some patients underwent surgical resection at an early stage. ICIs mainly included sintilimab, camrelizumab, pembrolizumab and duvalumab, etc. Treatment was administered every 3 weeks until disease progression or discontinuation for other reasons (e.g., intolerable side effects or death from another disease). Immunotherapy combined with chemotherapy was defined as at least 4 cycles of chemotherapy combined with immunotherapy if the patient did not have disease progression or discontinued the drug for other reasons (6).

### 2.3 ^18^F-FDG PET/CT acquisition

All patients were required to fast strictly for at least 6 hours before undergoing ^18^F-FDG PET/CT, to ensure the fasting blood glucose level could be maintained below 7.0mmol/L before ^18^F-FDG PET/CT injection. Weight, height, and blood glucose levels were measured before the imaging agent was injected. If the patient cannot cooperate well during the examination, the corresponding sedation method can be taken if necessary. The injection dose of ^18^F-FDG was 3.7-4.4MBq/kg. Patients were required to rest for 60 ± 10 minutes after injection of the imaging agent and were instructed to empty the bladder before imaging. The study involved using two PET/CT devices for scanning: GE Discovery Elite (Device 1) and GE Discovery ST4 (Device 2). The patient lies on their back with both arms raised. The scanning range is usually from the top of the head to the middle of the thighs. If the clinical suspicion or known lesion extends beyond this range, the scanning area should be appropriately expanded. First, low-dose CT scans were performed for attenuation correction of the images (using automatic tube current modulation technology, with tube voltage at 120 kV and slice thickness of 3 mm). Subsequently, three-dimensional PET scans were conducted (with 6–8 beds, each bed taking 2 minutes, with an increment of 16.2 cm, the exact number of beds being adjusted according to the patient’s height). The PET images were obtained using the VUE pointFX software, which integrates the ordered subset expectation maximization algorithm, time-of-flight information, and point spread function The cutoff point of the filter in Setup 1 is 6.4 millimeters, with 2 iterations and 24 subsets. The filter cutoff point of Equipment 2 is 5.0 millimeters, with 3 iterations and 16 subsets.

### 2.4 ^18^F-FDG-PET/CT image and biological analyses

**Figure 1:**
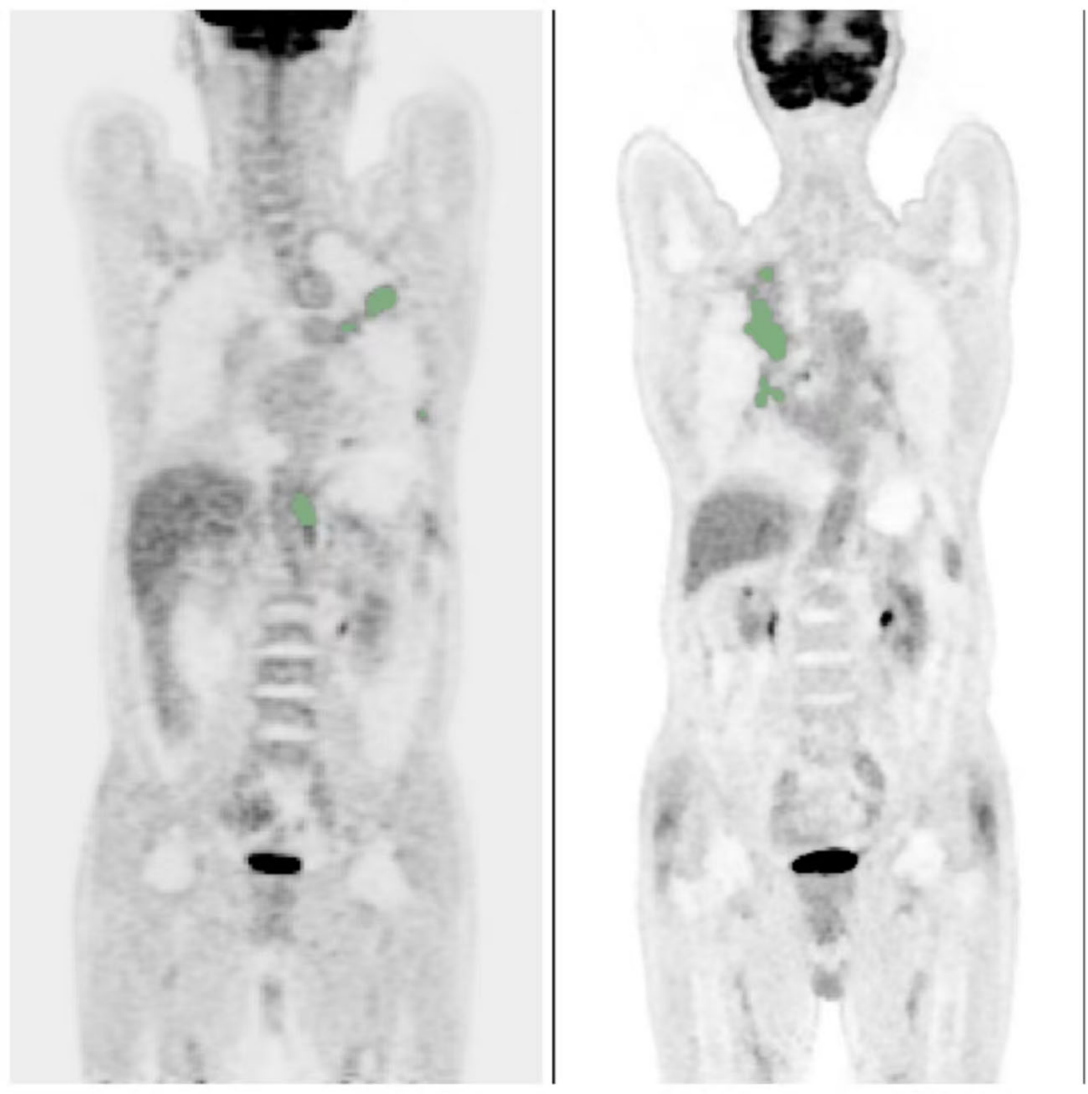
Delineation of whole-body metabolic tumor volume. NOS: Maximum intensity projection (MIP) images of an ^18^F-FDG PET/CT scan in a patient with NSCLC. The volumetric regions of interest encompassing all metastatic lesions were automatically delineated using a fixed SUV threshold of 2.5, and then manually adjusted by physicians. The total volume of these VOIs represents the MTVwb.

Two nuclear medicine physicians with senior medical qualifications used the 3D slicer software (version 5.2.2) to set the SUV (Standardized Uptake Value) as 2.5 as the threshold to initially screen out all the suspicious metabolically active volumes (Volumes of Interest, VOIs) that were higher than this threshold. The analysis was performed on 3D slicer software (version 5.2.2) (**Figure 1**). The two physicians then independently and separately compared these VOIs with the CT images from the same machine, excluded physiological uptake (such as the intestines, muscles, and brown fat) and inflammatory lesions based on the anatomical location, and confirmed all tumor-related lesions (primary lesions, metastatic lymph nodes, and distant metastases). For patients with liver metastases, the Volume of Interest (VOI) delineation standard for liver metastases is SUV ≥ SUVmean ± 3sd, and the lesion volume ≥ 1ml. Additionally, all lesions are semi-automatically delineated based on 40% of the lesion’s SUVmax as the threshold as the final VOI, which is used for obtaining metabolic parameters. The consistency of the two physicians in lesion delineation is evaluated by ICC, and the disagreement is resolved by the third nuclear medicine physician with over 10 years of experience for reaching a consensus. The 3D slicer software (version 5.2.2) measures and calculates the SUVmax, SUVmean, MTV, TLG, MTVwb and TLGwb of the primary lesion. MTVwb is obtained by summing up the MTVs of all tumor-related hotspots (the primary lesion, involved lymph nodes and metastatic foci), and TLGwb = MTVwb × the mean value of all tumor-related voxels. The receiver operating characteristics (ROC) curve was drawn, and the coordinate points on the ROC curve were used to calculate the Youden index. Youden index = sensitivity - (1- specificity). The optimal cut-off value of PET metabolic parameters was obtained. The patient’s electronic records (within 28 days before the first treatment) were reviewed, and baseline complete blood counts and lactate dehydrogenase (LDH) were obtained. For dNLR, dNLR=3 was determined as the best cut-off value for predicting prognosis according to the relevant studies on immune checkpoint inhibitors published so far (13, 14). For LDH, we used the upper limit of normal of our institute (LDH: ULN = 250Ul/L).

### 2.5 PD-L1 expression (TPS)

Tumor PD-L1 gene expression was evaluated by immunohistochemical analysis in pre-treatment surgical resection or biopsy specimens. According to a previous study (15–17), the Tumor Proportion Score (TPS) of PD-L1 gene expression was divided into two groups with a cut-off value of 50%, as follows: TPS≥50% was defined as “high PD-L1 expression”, and TPS<50% was defined as “low PD-L1 expression”, in which “low PD-L1 expression” included those without PD-L1 expression.

### 2.6 Outcomes: survival (OS and PFS) and disease clinical benefit

The data review deadline for this study is July 1, 2024. All patient follow-ups were conducted through telephone interviews. The clinical outcomes were disease clinical benefit (DCB), progression-free survival (PFS), and overall survival (OS). Patients underwent contrast CT scans every 6–8 weeks to assess response according to RECIST1.1, the reference standard for patient evaluation (18). Combined with RECIST1.1 and the treatment method of clinicians (without changing immune checkpoint inhibitors), patients with objective partial or complete response (PR or CR) at any time during treatment or stable disease (SD) after 6 months were considered to have disease clinical benefit.

### 2.7 Statistical analysis

All variables included in the analysis of this study had no missing data, so all analyses were based on the complete case set. In addition to the parameters discussed above for which predefined thresholds have been determined, the mean value was used as the cut-off value for the clinical parameter variables for those with normal distribution and the median value for those with non-normal distribution. Chi-square test was used for univariate analysis to analyze the correlation between independent variables and DCB. Factors with statistical significance (*p<*0.05) in univariate analysis were included in multivariate binary logistics regression analysis. For the clinical outcomes of OS and PFS, the COX proportional hazards regression model was used for univariate analysis. Factors with statistically significant (*p<*0.05) in univariate analysis were used for multivariate COX regression analysis in a stepwise manner to obtain their related independent influencing factors. The Kaplan–Meier method was used to draw the survival curve of independent influencing factors of clinical outcomes, and the median or mean of PFS and OS in each group was calculated. In our study, *p<*0.05 was considered statistically significant. The Chi-square Test was used for categorical variables, the Mann–Whitney test was used for univariate analysis of continuous variables, and binary logistics regression was used for multivariate analysis to obtain the independent influencing factors of PD-L1 expression. SPSS27.0 software was used to analyze the data.

## 3 Results

### 3.1 Patient characteristics

**Figure 2:**
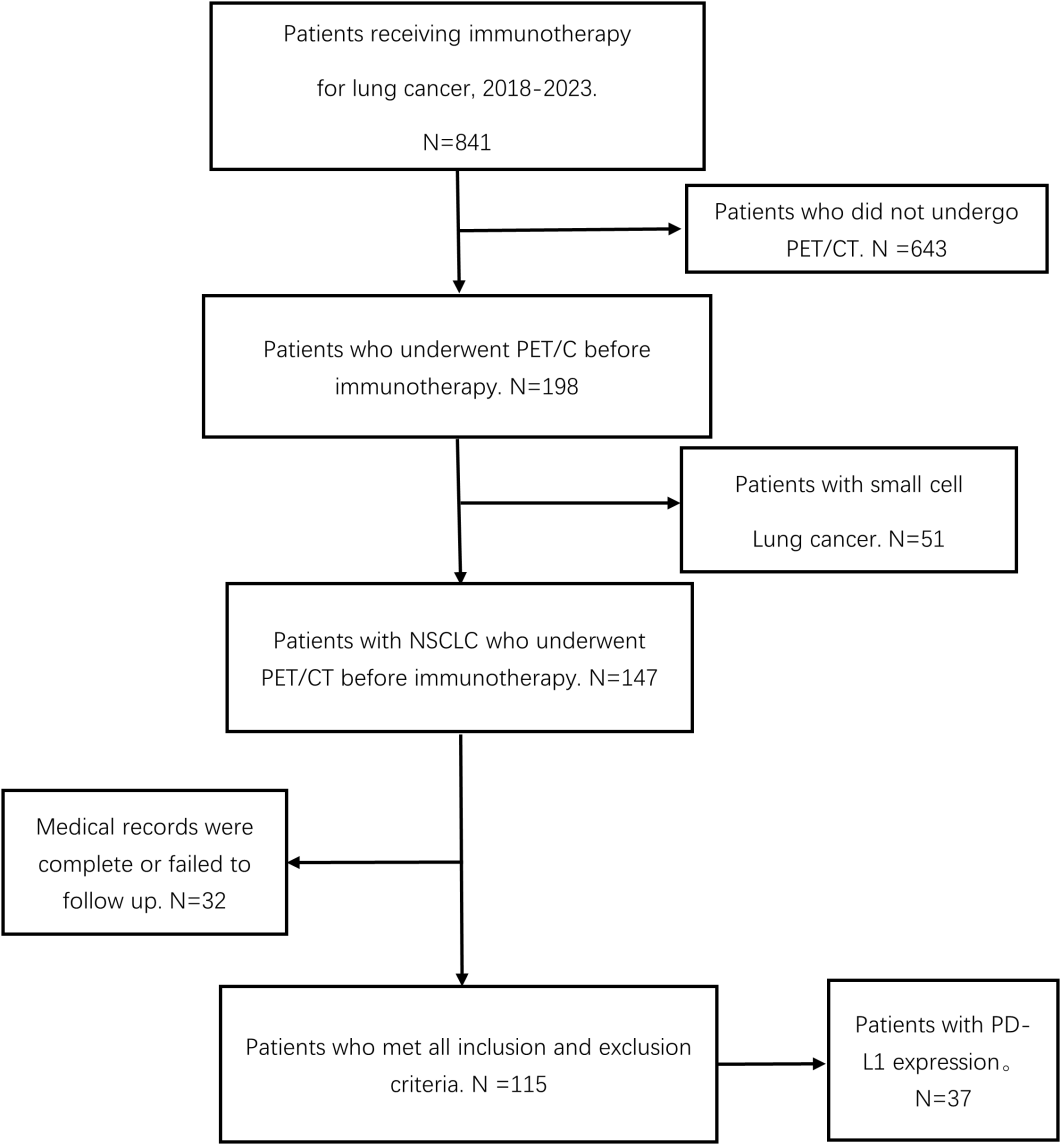
Flow chart of patient screening.

A total of 115 patients with advanced NSCLC were enrolled (**Figure 2**). The baseline clinical characteristics, hematological parameters, and PET metabolic parameters of the patients were summarized in **Tables 1**, **2**. The median age of the whole group was 65 years (63.45 ± 0.79). Among the patients, 66 cases (57.39%) were diagnosed with squamous cell carcinoma, 47 cases (40.87%) with adenocarcinoma, and 2 cases (1.47%) with sarcomatous carcinoma. All patients received ICIs treatment, of which 95 patients (82.61%) received ICIs treatment combined with chemotherapy. The median scan-to-treatment interval in this study was 16 days (interquartile range: 10–23 days; range: 0–28 days). A total of 32 patients with ICIs treatment combined with chemotherapy were detected with PD-L1 gene expression by immunohistochemistry, of which 12 patients (37.5%) had high expression. Most patients had a hematological examination 1 week before treatment, most often with an interval of less than 28 days.

**Table 1:** Overall population (N = 115) characteristics.

Characteristics	N	(n%)
Sex		
Male	102	88.70%
Female	13	11.30%
Age(years)		
<65	53	46.08%
≥65	62	53.91%
Smoking history		
Smoking	80	69.56%
Non-Smoking	35	30.43%
Histological variant (NSCLC)		
Squamous	66	57.39%
Non-squamous	49	42.61%
Stage		
III	31	26.96%
IV	84	73.04%
BMI		
≤25	55	47.83%
>25	60	52.17%
Treatment		
I+C	95	82.61%
I	20	17.39%
Immunotherapy		
First line	106	92.17%
≥Second line	9	7.83%

BMI, Body Mass Index; I, Immune Checkpoint Inhibitors; I+C, Immune checkpoint inhibitors plus chemotherapy.

**Table 2:** Patient’s characteristics according to ^18^F-FDG uptake and hematology parameters.

Characteristics	Mean ± SD	Median(range)
Biology		
dNLR	2.59 ± 1.44	2.23(0.24-11.55)
LDH(u/l)	238.39 ± 7.00	226.00(122.00-707.00)
PET Imaging Characteristics		
SUVmax	16.30 ± 9.35	16.03(0.00-69.81)
SUVmean	9.37 ± 5.54	9.72(0.00-40.54)
MTV(cm³)	45.39 ± 70.55	21.74(0.00-484.73)
TLG(cm³)	412.08 ± 561.95	245.1(0.00-3696.49)
MTVwb(cm³)	171.00 ± 170.65	112.24(1.55-848.01)
TLGwb(cm³)	1050.68 ± 1069.43	733.74(5.63-5716.12)

SD, standard deviation; dNLR, derived neutrophils to lymphocytes ratio; LDH, lactate dehydrogenase; SUVmax, maximum standardized uptake value; SUVmean, mean standardized uptake value; MTV, metabolic tumor volume; TLG, total lesion glycolysis; TLGwb, total lesion glycolysis of whole body; MTVwb, metabolic tumor volume of whole body.

### 3.2 DCB classification: patients with versus without clinical benefit

A total of 71 patients (61.7%) had clinical benefit. According to RECIST1.1, 62% of the patients achieved DCB after receiving PD-1/PD-L1 ICIs with/without chemotherapy (71 patients: 2 CP, 50 PR, and 19 SD>6 months). The metabolic tumor volume (MTV), the total lesion glycolysis (TLG) of primary lesions, the metabolic tumor volume of whole body (MTVwb), and the total lesion glycolysis of whole body (TLGwb) in DCB patients were significantly lower than those in non-DCB patients. Patients with immunotherapy combined with chemotherapy are more likely to achieve DCB. In binary logistics regression multivariate analysis, low MTVwb ([OR]3.91, 95%CI 1.64-9.28) and immunotherapy combined with chemotherapy ([OR]8.11,95%CI 2.51-26.21) were still the independent influencing factors of DCB (**Table 3****).** This result is the same as the independent influencing factors of PFS. Hematological factors dNLR and LDH are not related to DCB.

**Table 3:** Univariate and multivariate analyses of the relationship between patient characteristics and disease clinical benefit.

Characteristics	DCB(n=71)	non-DCB(n=84)	P		OR (CI 95%)
			Univariate analysis	Multivariate analysis	
Age(years)					
<65	35(66.0%)	18(34.0%)	0.38	–	–
≥65	1(3.7%)	26(96.3%)		–	–
Sex					
Male	63(61.8%)	39(38.2%)	0.99	–	
Female	8(61.5%)	5(38.5%)		–	
Smoking history					
Smoking	52(65.0%)	28(35.0%)	0.28	–	
Non-Smoking	19(54.3%)	16(45.7%)		–	
BMI					
≤25	33(60.0%)	22(40.0%)	0.71	–	
>25	38(63.3%)	22(36.7%)		–	
Treatment					
I+C	66(69.5%)	29(30.5%)	<0.001	<0.001	8.11(2.51-26.21)
I	5(25.0%)	15(75.0%)			
Histological Variant(NSCLC)					
Squamous	23(52.3%)	21(47.7%)	0.24	–	–
Non-squamous	45(68.2%)	21(31.8%)		–	–
Stage					
III	23(74.2%)	8(25.8%)	0.095	–	–
IV	48(57.1%)	36(42.9%)		–	–
dNLR					
≤3			0.966	–	–
>3				–	–
LDH(u/l)					
≤250	57(64.8%)	31(35.2%)	0.227	–	–
>250	14(51.9%)	13(48.1%)		–	–
SUVmax					
≤14.59	26(55.3%)	21(44.7%)	0.239	–	–
>14.59	45(66.2%)	23(33.8%)		–	–
SUVmean					
≤9.17	28(54.9%)	23(45.1%)	0.178	–	–
>9.17	43(67.2%)	21(32.8%)		–	–
MTV (cm³)					
≤39.51	53(69.7%)	23(30.3%)	0.014	–	–
>39.51	18(46.2%)	21(53.8%)		–	–
TLGvb(cm³)					
≤361.07	50(68.5%)	23(31.5%)	0.015	–	–
>361.07	21(50.0%)	21(50.0%)		–	–
MTVwb(cm³)					
≤122.10	45(75.0%)	15(25.0%)	0.002	0.002	3.906(1.644-9.279)
>122.10	26(47.3%)	29(52.7%)			
TLGwb(cm³)					
≤535.41	35(74.5%)	12(25.5%)	0.02	–	–
>535.41	36(52.9%)	32(47.1%)		–	–

BMI, Body Mass Index; I, Immune Checkpoint Inhibitors; I+C, Immune checkpoint inhibitors plus chemotherapy; dNLR, derived neutrophils to lymphocytes ratio; LDH, lactate dehydrogenase; SUVmax, maximum standardized uptake value; SUVmean, mean standardized uptake value; MTV, metabolic tumor volume; TLG, total lesion glycolysis; TLGwb, total lesion glycolysis of whole body; MTVwb, metabolic tumor volume of whole body; OR, odds ratio; CI, confidence interval.

### 3.3 Univariate and multivariate analyses: progression-free survival and overall survival

**Figure 3:**
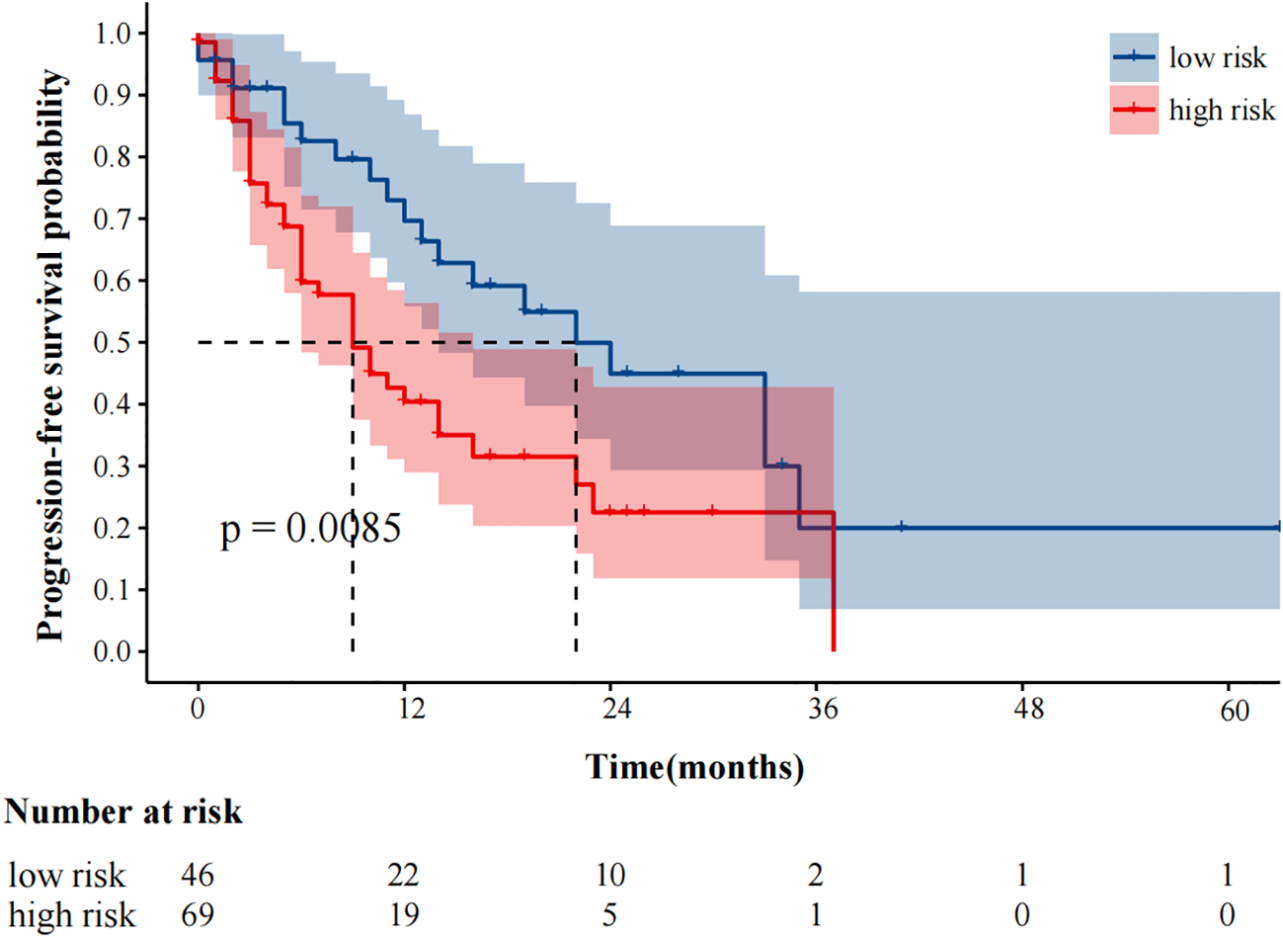
Kaplan-Meier plot analysis for MTVwb with PFS. NOS: low risk: MTVwb ≤ 90.81 cm³; high risk: MTVwb>90.81 cm³.

**Figure 4:**
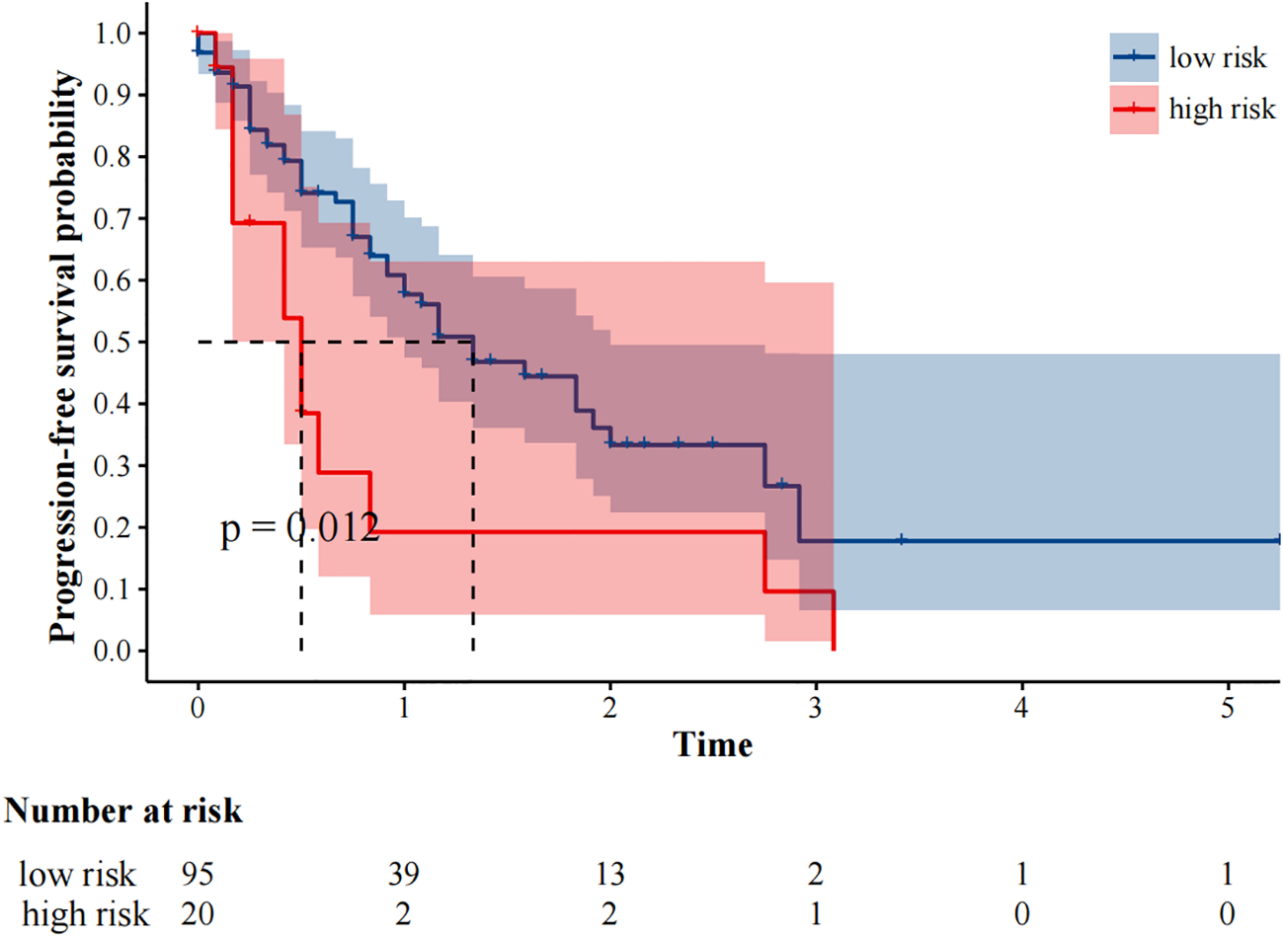
Kaplan-Meier plot analysis for treatment with PFS. NOS: low risk: Immune checkpoint inhibitors plus chemotherapy; high risk: Immune Checkpoint Inhibitors.

The median follow-up was 25 months. 59 (51.3%) and 48 (41.7%) patients experienced progression and died, respectively. The median progression-free survival was 14.0 (95%CI9.49–18.51) months. In our study, fewer than 50% of deaths occurred at the time of the cutoff for follow-up, and the median overall survival could not be calculated. In univariate analysis, immunotherapy alone, high MTV, TLG, MTVwb, and TLGwb were significantly associated with PFS (*p* < 0.05). In multivariate analysis, only immunotherapy alone (HR2.05, 95%CI1.07-3.90) and high MTVwb (HR 2.04, 95%CI1.17-3.55) were independently statistically significant parameters associated with poor PFS (**Table 4****).** K-M survival curve was drawn (**Figures 3**, **4**). The median PFS of immunotherapy alone and immunotherapy combined with chemotherapy were 6 months and 16 months, respectively. The median PFS was 9 months in the high MTVwb group and 22 months in the low MTVwb group. The median PFS was 9 months in the high MTVwb group and 22 months in the low MTVwb group. To assess the impact of the treatment method on the prognosis value of MTVwb, we conducted an interaction test on two factors. The results showed that the prognosis value of MTVwb for PFS (*p* = 0.356) was consistent in the monotherapy group and the combined treatment group.

**Table 4:** Prognostic significance of biomarkers for progression-free survival in univariate and multivariate analyses.

Variable	Total patients	Adverse event	Progression-free survival			
			Univariate analysis		Multivariate analysis	
	N (%)	N (%)	HR (95%CI)	P	HR (95%CI)	P
Age(≤65years)	61(53.0%)	27(50.9%)	1.060(0.629-1.787)	0.826	-	-
Sex(male)	102(88.7%)	48(47.1%)	1.727(0.688-4.335)	0.244	-	-
History(smoking)	80(69.6%)	43(53.8%)	0.758(0.446-1.287)	0.305	-	-
BMI (≤25)	55(47.8%)	26(47.3%)	1.185(0.706-1.989)	0.521	-	-
Histology (SCC)	66(57.4%)	58(42.4%)	1.391(0.815-2.375)	0.226	-	-
Stage (III)	31(27.0%)	14(45.2%)	0.968(0.548-1.712)	0.912	-	-
Treatment Line (1)	106(92.2%)	53(50.0%)	1.229(0.552-2.892)	0.636		
Treatment(I)	20(17.4%)	7(35.0%)	2.166(1.150-4.077)	0.017	2.045(1.073-3.896)	0.030
dNLR (>3)	29(25.2%)	12(41.4%)	1.099(0.578-2.087)	0.774	-	-
LDH (>250)	27(23.5%)	13(48.1%)	0.992(0.544-1.809)	0.978	-	-
SUVmax (>17.43)	52(45.2%)	22(42.3%)	1.316(0.788-2.197)	0.294	-	-
SUVmean (>11.43)	43(37.4%)	17(39.5%)	1.422(0.849-2.380)	0.181	-	-
MTV (>120.32 cm³)	60(52.2%)	25(41.7%)	1.910(1.126-3.239)	0.016	-	-
TLG (>434.15 cm³)	37(32.2%)	15(40.5%)	1.828(1.070-3.123)	0.027	-	-
MTVwb (>90.81 cm³)	69(60.0%)	30(43.5%)	2.041(1.173-3.552)	0.012	2.041(1.173-3.552)	0.012
TLGwb (>516.70 cm³)	69(60.0%)	31(44.9%)	1.843(1.068-3.181)	0.028	-	-

Total number of events in the entire cohort: 59/115; median progression-free survival: 14.0 months.

A total of 59 cases of progression-free survival events occurred in the entire cohort.

BMI, Body Mass Index; SCC, squamous cell carcinoma; I, Immune Checkpoint Inhibitors; dNLR, derived neutrophils to lymphocytes ratio; LDH, lactate dehydrogenase; SUVmax, maximum standardized uptake value; SUVmean, mean standardized uptake value; MTV, metabolic tumor volume; TLG, total lesion glycolysis; TLGwb, total lesion glycolysis of whole body; MTVwb, metabolic tumor volume of whole body; HR, hazard-ratio; CI, confidence interval.

**Figure 5:**
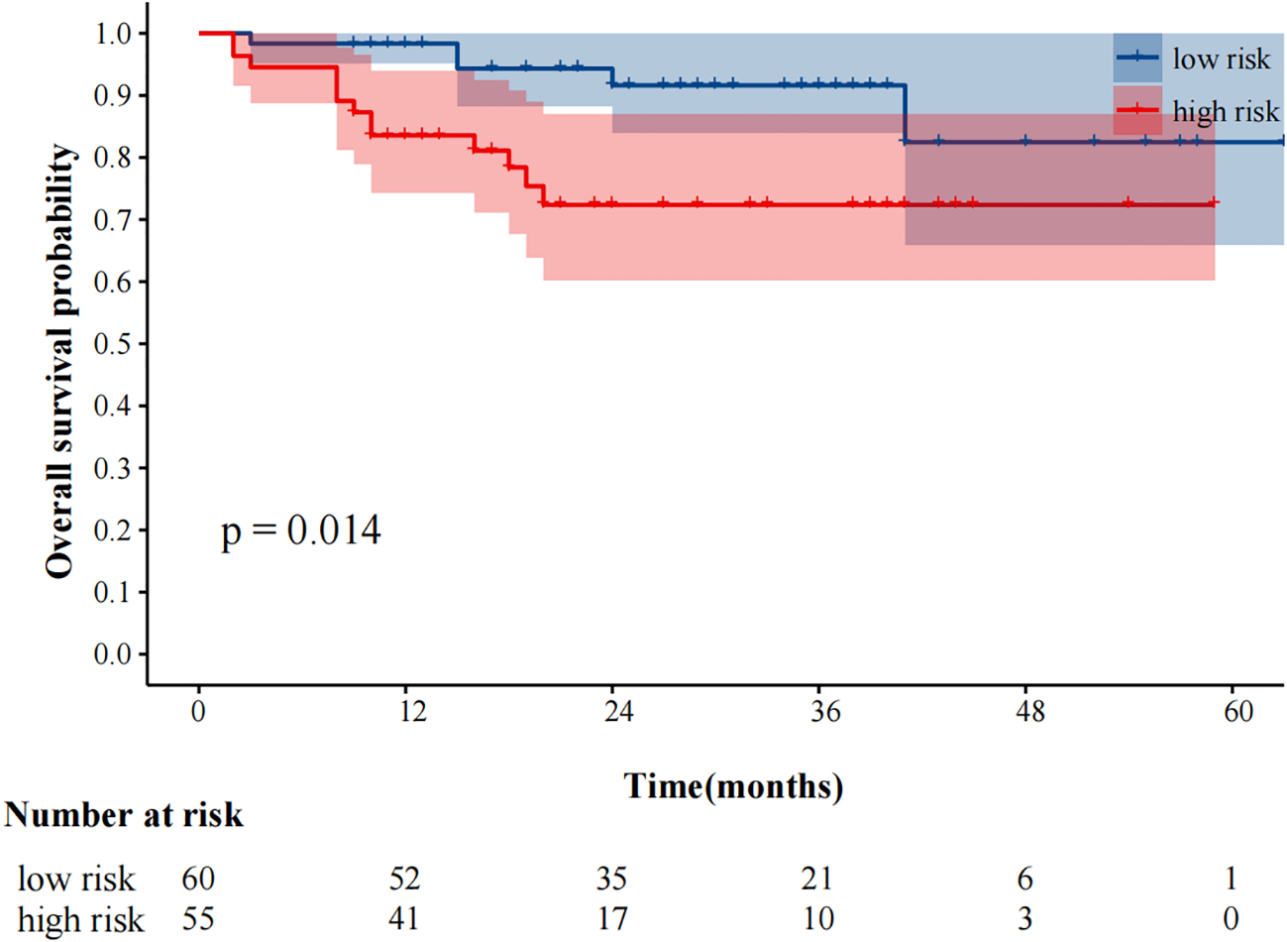
Kaplan-Meier plot analysis for MTVwb with OS. NOS: low risk: MTVwb ≤ 105.79 cm³; high risk: MTVwb>105.79 cm³.

In univariate analysis, SUVmax, MTV, TLG, MTVwb, and TLGwb were all significantly associated with OS (*p* < 0.05), and in multivariate analysis, only high MTVwb (HR4.173, 95%CI 1.36-12.84) was an independent factor for poor OS (**Table 5****).** The K-M survival curve was drawn (**Figure 5****).** The mean OS of high TLGwb and low TLGwb was 26 and 52 months, respectively.

**Table 5:** Prognostic significance of biomarkers for overall survival in univariate and multivariate analyses.

N=115	Overall survival			
	Univariate analysis		Multivariate analysis	
Variable	HR (95%CI)	P	HR (95%CI)	P
Age(≤65years)	1.081(0.612-1.909)	0.788	-	-
Sex(male)	1.288(0.486-3.102)	0.665	-	-
Smoking history(smoking)	1.505(0.767-2.952)	0.235	-	-
BMI (≤25)	0.747(0.423-1.318)	0.314	-	-
Histology (SCC)	1.059(0.389-2.886)	0.911	-	-
Treatment Line (1)	1.569(0.208-1.860)	0.662	-	-
Stage (III)	0.832(0.439-1.577)	0.573	-	-
Treatment(I)	1.486(0.758-2.915)	0.249	-	-
dNLR (>3)	1.701(0.921-3.142)	0.09	-	-
LDH (>250 u/l)	1.293(0.684-2.446)	0.429	-	-
SUVmax (>12.32)	2.458(1.188-5.084)	0.015	-	-
SUVmean (>11.82)	1.457(0.823-2.579)	0.197	-	-
MTV (>24.49 cm³)	3.157(1.729-5.764)	<0.001	-	-
TLG (>305.71 cm³)	2.828(1.572-5.087)	<0.001	-	-
MTVwb (>105.79 cm³)	5.009(2.534-9.901)	<0.001	4.173(1.356-12.842)	0.013
TLGwb (>918.25 cm³)	4.428(2.381-8.235)	<0.001	-	-

BMI, Body Mass Index; SCC, squamous cell carcinoma; I, Immune Checkpoint Inhibitors; dNLR, derived neutrophils to lymphocytes ratio; LDH, lactate dehydrogenase; SUVmax, maximum standardized uptake value; SUVmean, mean standardized uptake value; TLGwb, total lesion glycolysis of whole body; MTVwb, metabolic tumor volume of whole body; HR, hazard-ratio; CI, confidence interval.

### 3.4 PD-L1 expression: patients with PD-L1 positive versus negative tumor

All patients underwent immunohistochemical testing. But a total of 37 patients (32.2%) had PD-L1 genetic testing results. Compare the baseline data of patients who underwent PD-L1 testing with those who did not (**Table 6**). The results did not show any significant differences in baseline characteristics. The high deletion rate of PD-L1 gene testing is mainly due to the fact that the guidelines for the treatment of immune checkpoint inhibitors (19) do not require PD-L1 gene testing. For some patients, genetic testing was not performed in the hospital, and data were missing. A total of 32 patients in the ICIS combined with chemotherapy subgroup (N = 95) had PD-L1 gene detection, while only 5 patients in the immunotherapy alone group had PD-L1 gene detection records, so only the data of patients with ICIs combined with chemotherapy were statistically analyzed. The statistical analysis of the correlation between clinical characteristics, hematological parameters, and PET metabolic parameters in patients with high PD-L1 and low PD-L1 gene expression (TPS≥50% and TPS<50%, respectively) is shown in **Table 7** There was a significant correlation between PD-L1 expression and TLGwb (*p* =0.029).

**Table 6:** Comparison of baseline characteristics between patients with and without PD-L1 testing.

Characteristics	All patients (N = 115)	Testing group (N = 37)	Non-tested group (N = 78)	P
Age(years)	65(28-79)	65 (28-76)	65(45-79)	0.701
Sex				1.000
Male	102 (88.7)	33 (89.2)	69 (88.5)	
Female	13 (11.3)	4 (10.8)	9 (11.5)	
Smoking history				0.105
Smoking	80 (69.6)	22 (59.5)	58 (74.4)	
Non-Smoking	35 (30.4)	15(40.5)	20 (25.6)	
BMI	25(3.71)	25(3.31)	26(3.87)	0.184
Treatment				0.450
I+C	20(17.4)	5(13.5)	15(19.2)	
I	95(82.6)	32(86.5)	63(80.8)	
Stage				0.375
III	31(27.0)	8(21.6)	23(29.5)	
IV	84(73.0)	29(78.4)	55(70.5)	
Histological variant (NSCLC)				0.012
Squamous	66(57.4)	15(40.5)	51(65.4)	
Non-squamous	49(42.6)	22(59.5)	27(34.6)	

BMI, Body Mass Index; I, Immune Checkpoint Inhibitors; I+C, Immune checkpoint inhibitors plus chemotherapy.

**Table 7:** Univariate and multivariate analyses of the relationship between patient characteristics and PD-L1 expression level.

n=37	PD-L1 expression level				OR (CI 95%)
Variable	≥50%(n=12)	<50%(n=20)	p		
			Univariate Analysis	Multivariate Analysis	
Age(years)					
<65	5	9	0.625	-	-
≥65	7	11		-	-
Sex					
Male	12	21	0.625	-	-
Female	2	2		-	-
Smoking history					
Smoking	9	12	0.387	-	-
Non-Smoking	3	8		-	-
BMI					
≤25	4	16	0.710	-	-
>25	8	4		-	-
Histological variant(NSCLC)					
Squamous	3	9	0.240	-	-
Non-squamous	9	11		-	-
Stage					
vIII	10	16	0.815	-	-
IV	2	4		-	-
dNLR					
≤3	7	16	0.966	-	-
>3	5	4		-	-
LDH(u/l)					
≤250	5	15	0.059	-	-
>250	7	5		-	-
SUVmax	-	-	0.138	-	-
SUVmean	-	-	0.130	-	-
MTV	-	-	0.588	-	-
TLG	-	-	0.101	-	-
MTVwb	-	-	0.175	-	-
TLGwb	-	-	0.031	0.029	1.001(1.000-1.002)

BMI, Body Mass Index; dNLR, derived neutrophils to lymphocytes ratio; LDH, lactate dehydrogenase; SUVmax, maximum standardized uptake value; SUVmean, mean standardized uptake value; TLGwb, total lesion glycolysis of whole body; MTVwb, metabolic tumor volume of whole body; HR, hazard-ratio; CI, confidence interval.

## 4 Discussion

PET/CT imaging, as a fusion imaging of CT and PET, can provide anatomical structure information and molecular metabolic information of lesions at the same time, so as to find metastatic lesions more sensitively and evaluate the disease more comprehensively. Previous studies have confirmed that tumor burden and metabolic changes are associated with the prognosis of patients receiving PD-1 inhibitors combined with chemotherapy. The aim of this study is to explore the predictive value of ^18^F-FDG PET metabolic parameters, including PET metabolic parameters of primary lesions and systemic lesions, in the survival prognosis of advanced NSCLC patients treated with PD-1/PD-L1 inhibitors with/without chemotherapy. The results show that patients with low MTVwb are more likely to benefit from the treatment and achieve longer PFS and OS. The advantage of this study lies in the extremely high completeness of data for all variables, with no missing values. This avoids potential biases that may arise from data interpolation and enhances the strength of the statistical conclusions. Furthermore, we found that the treatment method does not affect the prognostic effect of MTVwb. MTVwb is a reliable prognostic biomarker, and its value is not influenced by the changes in the treatment strategies covered by this analysis. Spearman correlation analysis in this study demonstrated a very strong positive correlation between MTVwb and TLGwb (rs = 0.956, P < 0.001). However, the multivariate model ultimately confirmed that only MTVwb was an independent prognostic factor. Therefore, we did not conduct principal component analysis to preserve the clear clinical interpretability of MTVwb. In future studies involving multiple significant imaging parameters, it is advisable to consider using dimensionality reduction techniques such as PCA to construct composite indicators. A retrospective study by Monaco Let al. (20) included 92 lung cancer patients treated with PD-1/PD-L1 monotherapy and found that the baseline PET metabolic parameter MTVwb was an independent predictor of ICIs treatment response. In some other studies, which metabolic parameters have higher specificity and sensitivity and higher predictive value still need to be explored by prospective studies with large sample sizes. Some studies (18, 21, 22) also hold that PET metabolic parameters MTVwb/TMTV or primary tumor metabolic volume MTV and total lactate production (TLG) are valuable indicators for predicting the outcome and survival period of immunotherapy in NSCLC patients. Some studies also suggest that SUVmax can also predict the prognosis of lung cancer immunotherapy (23), but in recent years, many studies on lung cancer immunotherapy have shown that tumor metabolic volume and total glycolysis seem to be better than semi-quantitative parameters (such as SUVmax) to reflect the prognosis. However, which metabolic parameters have higher specificity and sensitivity and higher predictive value still need to be explored in prospective studies with large sample sizes. At present, immunotherapy combined with chemotherapy has become an important strategy for the first-line treatment of advanced NSCLC. This study also included patients who received immunotherapy as a single-agent treatment and those who received immunotherapy in combination. The results show that the combined treatment group performed significantly better than the single-drug treatment group in terms of prognosis. This discovery provides new evidence-based basis and clinical ideas for formulating more precise and personalized treatment plans for patients with advanced NSCLC. This might be related to the mechanism of action of chemotherapy. Not only can it inhibit the growth and proliferation of tumor cells through multiple mechanisms, but it can also act on various immune cells in the body (24). By blocking the activity of immunosuppressive cells, such as regulatory T cells, myeloid suppressor cells, and tumor-associated macrophages, or directly inducing the upregulation of MHC1 class molecules expression and the maturation of dendritic cells, it can promote the anti-tumor immune response of the body, thereby achieving a synergistic effect in tumor immunotherapy. In a meta-analysis by Zhou, Yixin et (25), 5 clinical trials with a total of 1289 patients were included for subgroup analysis of PD-L1≥50%. They also agreed that the objective response rate (ORR) and PFS of immunotherapy plus chemotherapy were superior to immunotherapy alone, and there was no significant difference in OS. At the same time, another meta-analysis (26)including 11 randomized controlled trials with a total of 6731 patients, analyzed the subgroup of patients with PD-L1≥50%. They also agreed that immunotherapy plus chemotherapy was superior to immunotherapy alone in terms of PFS (HR = 0.54, 95%CI: 0.35-0.82), but OS (HR = 0.86,95%CI: 0.65-1.14) was not significant. The results of these two studies were similar to those of the present study, but subgroup analysis of PD-L1 expression was not performed due to the small sample size of PD-L1 gene expression in this article. This outcome leads to an increased risk of committing a Type II error. Meanwhile, patients who undergo PD-L1 testing may be influenced by the specific clinical criteria of this research center or the judgment of the doctors. This may lead to selection bias. In the future, we will continue to increase the sample size and conduct multi-center data collection, in order to obtain more widely applicable research results. At present, there are more and more studies on PET/CT parametric radiomics to predict the efficacy of immunotherapy. Mu, Wei et (27) used PET/CT radiomics to predict the durable clinical benefit of immunotherapy, which proved the good prospect of multi-parameter radiomics features (mpRS) in predicting the durable clinical benefit. With the emergence of many related studies on imaging parameters and radiomics, the number of image features extracted from PET/CT parameters is increasing, which may cause redundancy and lack of reproducibility of image features (28). Therefore, how to select image feature parameters more suitable for clinical practice becomes very important.

dNLR encompasses monocytes and other granulocyte subsets and can capture systemic inflammatory responses as well as changes in peripheral blood leukocytes. Zhang Lei et al. (29). However, in this study, the hematological parameter dNLR did not show any statistically significant difference related to prognosis. Taking into account that this study and the studies by Zhang Lei et (29) have potential sample selection errors due to the inclusion of advanced NSCLC patients, and that the PD-1 inhibitors used and the chemotherapy regimens are different. In this study, the same sample size was used to conduct parallel analyses of hematological and PET metabolic parameters. A scientific and rigorous head-to-head comparison was employed to ensure the fairness and reliability of the research results. This method has significantly promoted the development of efficacy prediction research for ICIs toward greater precision, practicality and cost-effectiveness. But it is necessary to conduct multi-center large-sample prospective related studies in the future to deeply analyze this issue.

In this study, patients with high expression of the PD-L1 gene also showed a significant increase in TLGwb. Evangelista et al. (31) suggested that the high expression of PD-L1 was also associated with TLGwb, SUVmax, and MTV. Moreover, the sample size of this study was relatively large, and the results were quite convincing. In NSCLC, PD-L1 gene expression is currently the only biomarker approved by FAD for the specific PD-1/PD-L1 inhibitor. However, in clinical practice, there are technical issues in PD-L1 detection (such as batch effects of staining itself) and biological reasons for PD-L1 (such as sampling errors caused by uneven distribution). Moreover, some patients are unable to obtain sufficient tumor tissues for PD-L1 staining. Therefore, some studies have further investigated the relationship between tumor glycolysis and PD-L1 expression and explored more favorable biomarkers.

This study is a retrospective single-center study with a relatively small sample size and a short follow-up period. Inevitably, the completeness of medical records and the accuracy of data are limited, which may lead to information bias. Although we have endeavored to minimize this bias through a rigorous data verification process, we will still be committed to conducting prospective studies in the future to confirm the reliability of this result. The use of glucocorticoids at baseline has been associated with adverse outcomes with immunotherapy in some studies (30, 31), but these patients were not excluded because of the small number of patients who received glucocorticoids in our study. The ongoing debate about the optimal TMTV measurement hinders comparability across studies. In this study, there are individual differences in the use of PD-1/PD-L1 inhibitors. These drugs have slightly different mechanisms of action in the microenvironment, but the clinical efficacy differences remain inconclusive for now. Although the iRECIST efficacy assessment criteria have been recommended for use in clinical trials. The RECIST1.1 standard may not be able to accurately identify all patients who benefit from immunotherapy. The study is a retrospective study; the follow-up period is quite long. At present, in the NCCN guidelines, the RECIST 1.1 standard is still regarded as the standard tool for evaluating the therapeutic efficacy of most solid tumors. the consensus guideline iRECIST was not used (31).

Our research further substantiates the ability of tumor metabolism to evaluate the therapeutic efficacy of ICIs treatment. Immune checkpoint inhibitors can correct the escape inhibition of T cells by the tumor microenvironment (TME) and restore T cells to a normal activated state. MTVwb is a powerful prognostic biomarker, which lays the foundation for its future clinical application. In the early stage of diagnosis, MTVwb can be used to identify patients with a high tumor burden and poor prognosis. However, its ability to predict the prognosis of immunotherapy can provide an early and precise diagnostic plan for these patients. One of the major challenges in truly transforming MTVwb into a clinical tool lies in the standardization and validation of its threshold. In this study, the cutoff values (such as the median or the optimal value determined by the ROC curve) were data-driven and originated from the analysis of the current retrospective cohort. Although it has significant statistical significance, it should be cautious to directly apply this result to other populations, as there is a risk of overfitting. Therefore, the results of this study urgently need to be verified through prospective, multi-center clinical trials. This is the necessary path to bring MTVwb into clinical application. The verification study we envision should include: 1. Prospective design, where the treatment methods for patients and the timing of PET/CT examinations are pre-defined, and data are collected in large, multi-center cohorts. 2. Standardized procedures: All participating centers are required to follow the unified standard operating procedures for PET/CT scans and image analysis to minimize technical variations. 3. Clinical efficacy verification: The ultimate goal of this study is not merely to reconfirm the prognostic value of MTVwb, but to construct an integrated model and create a new stratification index.

## 5 Conclusions

High MTVwb of baseline PET metabolic parameters is associated with poor PFS, OS, and non-DCB in advanced NSCLC patients treated with PD-1/PD-L1 inhibitors. Patients treated with ICIs combined with chemotherapy are more likely to achieve DCB and better PFS than those treated with ICIs alone, but it is not associated with OS.^18^F-FDG uptake may be a promising predictor of the response to PD-1/PD-L1 inhibitor therapy, and it may be useful for individualized treatment planning in clinical practice (31).

## Data Availability

The datasets presented in this study can be found in online repositories. The names of the repository/repositories and accession number(s) can be found in the article/supplementary material.

## References

[1] Global cancer burden growing, amidst mounting need for services. Saudi Med J. (2024) 45:326–7., PMID: 38438207 PMC11115397

[2] Abdurixiti M Nijiati M Shen R Ya Q Abuduxiku N Nijiati M . Current progress and quality of radiomic studies for predicting EGFR mutation in patients with non-small cell lung cancer using PET/CT images: a systematic review. Brit J Radiol. (2021) 94:20201272. doi: 10.1259/bjr.20201272, PMID: 33882244 PMC8173688

[3] Robert C . A decade of immune-checkpoint inhibitors in cancer therapy. Nat Commun. (2020) 11:3801. doi: 10.1038/s41467-020-17670-y, PMID: 32732879 PMC7393098

[4] Seban RD Mezquita L Berenbaum A Dercle L Botticella A Le Pechoux C . Baseline metabolic tumor burden on FDG PET/CT scans predicts outcome in advanced NSCLC patients treated with immune checkpoint inhibitors. Eur J Nucl Med Mol I. (2020) 47:1147–57. doi: 10.1007/s00259-019-04615-x, PMID: 31754795

[5] Hashimoto K Kaira K Imai H Mouri A Shiono A Miura Y . Prognostic potential of metabolic activity on 18 F-FDG accumulation in advanced NSCLC receiving combining chemotherapy plus PD-1 blockade. J Immunother. (2022) 45:349–57. doi: 10.1097/CJI.0000000000000434, PMID: 35980360

[6] Horn L Mansfield AS Szczesna A Havel L Krzakowski M Hochmair MJ . First-line atezolizumab plus chemotherapy in extensive-stage small-cell lung cancer. New Engl J Med. (2018) 379:2220–9. doi: 10.1056/NEJMoa1809064, PMID: 30280641

[7] Paz-Ares LG Ramalingam SS Ciuleanu TE Lee JS Urban L Caro RB . First-line nivolumab plus ipilimumab in advanced NSCLC: 4-year outcomes from the randomized, open-label, phase 3 CheckMate 227 part 1 trial. J Thorac Oncol. (2022) 17:289–308. doi: 10.1016/j.jtho.2021.09.010, PMID: 34648948

[8] Consensus on the immunohistochemical tests of PD-L1 in solid tumors (2021 version). Zhonghua Bing Li Xue Za Zhi. (2021) 50:710–8. doi: 10.3760/cma.j.cn112151-20210228-00172, PMID: 34405603

[9] Gandara DR Paul SM Kowanetz M Schleifman E Zou W Li Y . Blood-based tumor mutational burden as a predictor of clinical benefit in non-small-cell lung cancer patients treated with atezolizumab. Nat Med. (2018) 24:1441–8. doi: 10.1038/s41591-018-0134-3, PMID: 30082870

[10] Galvano A Gristina V Malapelle U Pisapia P Pepe F Barraco N . The prognostic impact of tumor mutational burden (TMB) in the first-line management of advanced non-oncogene addicted non-small-cell lung cancer (NSCLC): a systematic review and meta-analysis of randomized controlled trials. ESMO Open. (2021) 6:100124. doi: 10.1016/j.esmoop.2021.100124, PMID: 33940346 PMC8111593

[11] Lang D Horner A Brehm E Akbari K Hergan B Langer K . Early serum tumor marker dynamics predict progression-free and overall survival in single PD-1/PD-L1 inhibitor treated advanced NSCLC-A retrospective cohort study. Lung Cancer. (2019) 134:59–65. doi: 10.1016/j.lungcan.2019.05.033, PMID: 31319996

[12] Kaira K Kuji I Kagamu H . Value of (18)F-FDG-PET to predict PD-L1 expression and outcomes of PD-1 inhibition therapy in human cancers. Cancer Imaging. (2021) 21:11. doi: 10.1186/s40644-021-00381-y, PMID: 33441183 PMC7805193

[13] Park W Kwon D Saravia D Desai A Vargas F El DM . Developing a predictive model for clinical outcomes of advanced non-small cell lung cancer patients treated with nivolumab. Clin Lung Cancer. (2018) 19:280–8. doi: 10.1016/j.cllc.2017.12.007, PMID: 29336998

[14] Mezquita L Auclin E Ferrara R Charrier M Remon J Planchard D . Association of the lung immune prognostic index with immune checkpoint inhibitor outcomes in patients with advanced non-small cell lung cancer. JAMA Oncol. (2018) 4:351–7. doi: 10.1001/jamaoncol.2017.4771, PMID: 29327044 PMC5885829

[15] Ettinger DS Wood DE Aisner DL Akerley W Bauman JR Bharat A . Non-small cell lung cancer, version 3.2022, NCCN clinical practice guidelines in oncology. J Natl Compr Canc Ne. (2022) 20:497–530. doi: 10.6004/jnccn.2022.0025, PMID: 35545176

[16] Reck M Rodriguez-Abreu D Robinson AG Hui R Csoszi T Fulop A . Five-year outcomes with pembrolizumab versus chemotherapy for metastatic non-small-cell lung cancer with PD-L1 tumor proportion score >/= 50. J Clin Oncol. (2021) 39:2339–49. doi: 10.1200/JCO.21.00174, PMID: 33872070 PMC8280089

[17] Zhang X Wu M Chen J Zheng K Du H Li B . Comparative efficacy of immune checkpoint inhibitors combined with chemotherapy in patients with advanced driver-gene negative non-small cell lung cancer: A systematic review and network meta-analysis. Heliyon. (2024) 10:e30809. doi: 10.1016/j.heliyon.2024.e30809, PMID: 38774326 PMC11107224

[18] Eisenhauer EA Therasse P Bogaerts J Schwartz LH Sargent D Ford R . New response evaluation criteria in solid tumours: revised RECIST guideline (version 1.1). Eur J Cancer. (2009) 45:228–47. doi: 10.1016/j.ejca.2008.10.026, PMID: 19097774

[19] Thompson JA Schneider BJ Brahmer J Achufusi A Armand P Berkenstock MK . Management of immunotherapy-related toxicities, version 1.2022, NCCN clinical practice guidelines in oncology. J Natl Compr Canc Ne. (2022) 20:387–405. doi: 10.6004/jnccn.2022.0020, PMID: 35390769

[20] Monaco L Gemelli M Gotuzzo I Bauckneht M Crivellaro C Genova C . Metabolic parameters as biomarkers of response to immunotherapy and prognosis in non-small cell lung cancer (NSCLC): A real world experience. Cancers. (2021) 13:7. doi: 10.3390/cancers13071634, PMID: 33915801 PMC8037395

[21] Kim CG Hwang SH Kim KH Yoon HI Shim HS Lee JH . Predicting treatment outcomes using (18)F-FDG PET biomarkers in patients with non-small-cell lung cancer receiving chemoimmunotherapy. Ther Adv Med Oncol. (2022) 14:17486532. doi: 10.1177/17588359211068732, PMID: 35035536 PMC8753071

[22] Zhou Y Lin Z Zhang X Chen C Zhao H Hong S . First-line treatment for patients with advanced non-small cell lung carcinoma and high PD-L1 expression: pembrolizumab or pembrolizumab plus chemotherapy. J Immunother Cancer. (2019) 7:120. doi: 10.1186/s40425-019-0600-6, PMID: 31053172 PMC6500047

[23] Liang H Liu Z Cai X Pan Z Chen D Li C . PD-(L)1 inhibitors vs. chemotherapy vs. their combination in front-line treatment for NSCLC: An indirect comparison. Int J Cancer. (2019) 145:3011–21. doi: 10.1002/ijc.32366, PMID: 31018251

[24] Burtness B Harrington KJ Greil R Soulieres D Tahara M de Castro GJ . Pembrolizumab alone or with chemotherapy versus cetuximab with chemotherapy for recurrent or metastatic squamous cell carcinoma of the head and neck (KEYNOTE-048): a randomised, open-label, phase 3 study. Lancet. (2019) 394:1915–28. doi: 10.1016/S0140-6736(19)32591-7, PMID: 31679945

[25] Mu W Tunali I Gray JE Qi J Schabath MB Gillies RJ . Radiomics of (18)F-FDG PET/CT images predicts clinical benefit of advanced NSCLC patients to checkpoint blockade immunotherapy. Eur J Nucl Med Mol I. (2020) 47:1168–82. doi: 10.1007/s00259-019-04625-9, PMID: 31807885 PMC8663718

[26] Berenguer R Pastor-Juan M Canales-Vazquez J Castro-Garcia M Villas MV Mansilla LF . Radiomics of CT features may be nonreproducible and redundant: influence of CT acquisition parameters. Radiology. (2018) 288:407–15. doi: 10.1148/radiol.2018172361, PMID: 29688159

[27] Li N Zheng X Gan J Zhuo T Li X Yang C . Effects of glucocorticoid use on survival of advanced non-small-cell lung cancer patients treated with immune checkpoint inhibitors. Chin Med J Peking. (2023) 136:2562–72. doi: 10.1097/CM9.0000000000002544, PMID: 37925595 PMC10617908

[28] Chan KK Bass AR . Impact of non-steroidal anti-inflammatory drugs, glucocorticoids, and disease-modifying anti-rheumatic drugs on cancer response to immune checkpoint inhibitor therapy. Rheum Dis Clin N Am. (2024) 50:337–57. doi: 10.1016/j.rdc.2024.02.007, PMID: 38670731

[29] Li C Wu J Jiang L Zhang L Huang J Tian Y . The predictive value of inflammatory biomarkers for major pathological response in non-small cell lung cancer patients receiving neoadjuvant chemoimmunotherapy and its association with the immune-related tumor microenvironment: a multi-center study. Cancer Immunol Immun. (2023) 72:783–94. doi: 10.1007/s00262-022-03262-w, PMID: 36056951 PMC10991885

[30] Houdek S Buchler T Kindlova E . Comparison of RECIST 1.1 and iRECIST for response evaluation in solid tumours. Klin Onkol. (2017) 30:32–9. doi: 10.14735/amko20173S32, PMID: 29239190

[31] Singla R Jajodia A Agrawal RK Rao A Pasricha S Batra U . Comparison of RECIST and iRECIST criteria in patients with advanced lung cancer treated with nivolumab. J Cancer Res Ther. (2023) 19:1212–8. doi: 10.4103/jcrt.jcrt_1456_21, PMID: 37787285

